# Protective Effect of Quercetin on Melphalan-Induced Oxidative Stress and Impaired Renal and Hepatic Functions in Rat

**DOI:** 10.1155/2014/936526

**Published:** 2014-12-11

**Authors:** Ebenezer Tunde Olayinka, Ayokanmi Ore, Olaniyi Solomon Ola, Oluwatobi Adewumi Adeyemo

**Affiliations:** Biochemistry Unit, Department of Chemical Sciences, Ajayi Crowther University, PMB 1066, Oyo, 211213 Oyo State, Nigeria

## Abstract

One major challenge with the use of anticancer agents is the phenomenon of drug-induced toxicity. Melphalan (MPLN) is an alkylating anticancer agent, while quercetin (QCT) is an antioxidant. We investigated the protective role of quercetin against MPLN-induced toxicity. Twenty-five male Wistar rats (160–170 g) were randomized into five treatment groups; (I) control, (II) MPLN (0.2 mg/kg b.w.), (III) pre-treated with QCT (20 mg/kg b.w.) for 7 days followed by MPLN (0.2 mg/kg b.w.) for 7 days, (IV) cotreated with QCT (20 mg/kg b.w.) and MPLN (0.2 mg/kg b.w.) for 7 days, and (V) QCT (20 mg/kg b.w.) alone. MPLN caused a significant increase in plasma bilirubin, urea, and creatinine by 122.2%, 102.3%, and 188%, respectively (*P* < 0.05). Similarly, plasma ALP, ALT, AST, and *γ*-GT activities increased significantly by 57.9%, 144.3%, 71.3%, and 307.2%, respectively, relative to control. However, pre or cotreatment with QCT ameliorated the levels of renal and hepatic function indices. Hepatic ascorbic acid and GSH and activities of glutathione-S-transferase, SOD, and catalase decreased significantly by 36.2%, 188%, 46.5%, 34.4%, and 55.2%, respectively, followed by increase in MDA content by 46.5% relative to control. Pre- and cotreatment with QCT reestablished the hepatic antioxidant status and lipid peroxidation. Overall, quercetin protected against MPLN-induced renal and hepatic toxicity in rats.

## 1. Introduction

**Figure 1:**
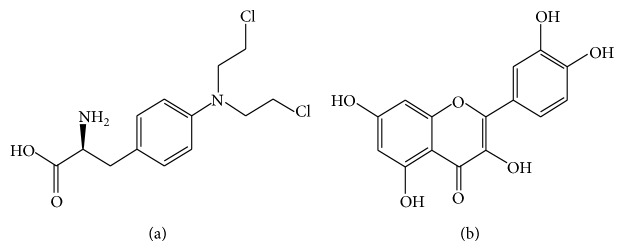
Structure of (a) melphalan and (b) quercetin.

Melphalan (4-[bis(2-chloroethyl)amino]-L-phenylalanine), Figure 1(a), is a phenylalanine derivative of nitrogen mustard. It is a bifunctional alkylating agent and one of the most aggressive antineoplastic drugs indicated for multiple myeloma and ovarian cancer [1]. Melphalan (MPLN) is classified as a cell cycle phase-nonspecific alkylating agent [2]. Its mechanism of action involves inhibition of DNA and RNA synthesis through formation of interstrand cross-links with DNA. Following oral administration, it is distributed mainly to the liver where microsomal glutathione-S-transferase (GST) plays a significant role in its metabolism [3]. Among the reported toxicities elicited by melphalan are hematological suppression [4], hepatotoxicity [5–7], renal toxicity [8, 9], and bone marrow suppression [10].

One of the cytotoxic mechanisms of alkylating agents is their ability to generate free radicals and trigger oxidative stress* in vivo* [11]. This may also be associated with the toxicities elicited by these drugs as a result of suppression of cellular antioxidant defense [12–14]. A free radical is a reactive atom or group of atoms that has one or more unpaired electrons. They are produced in the body by natural biological processes or introduced from an exogenous source such as drugs and environmental toxicants [15]. Excessive production of free radicals which are not neutralised may result in lipid, proteins, and DNA oxidation and ultimately in cell damage [15].

The liver is the main site of drug metabolism and metabolites generated in the liver and, in some cases, free drug molecules are also distributed to the kidneys, thus exposing these organs to drug-induced toxicities. These tissues have, however, evolved an array of antioxidant defense systems to protect against the harmful effect of drug metabolites and free radicals [15, 16]. Antioxidants are substances that inhibit oxidation or reactions promoted by oxygen, peroxides, or free radicals or their actions [16]. These include the nonenzymic antioxidants like reduced glutathione (GSH), ascorbic acid (AA), and vitamin E among others. Enzymic antioxidants involved in this protection include glutathione-S-transferase (GST), glutathione peroxidase (GPx), glutathione reductase (GR), superoxide dismutase (SOD), and catalase (CAT) [17, 18].

Quercetin (3,5,7,3′,4′-pentahydroxyflavone), Figure 1(b), is one of the most widely distributed flavonoids (of the flavonols subclass) in plants [19]. It is abundant in plants, plant products, and foods, such as red wine, onions, green tea, apples, broccoli, berries, ginkgo biloba, and buckwheat tea [20]. QCT has been exhibited in several studies as a potent antioxidant with a very strong free radical scavenging capacity [21, 22]. It also possesses a number of pharmacological activities including antidiabetic, anti-inflammatory, and immunostimulatory and protection of low density lipoprotein against oxidation [22–24]. Previous reports suggest that QCT also possesses the capacity to effectively inhibit the proliferation of cancer cells [25–27]. Moreover, it is known to improve chemotherapeutic efficacy of certain alkylating agents [28]. In addition, recent studies demonstrated that quercetin could effectively attenuate drug-induced toxicity and oxidative stress* in vivo* [29–31].

One of the major challenges often encountered with the use of anticancer agents is the incident of drug-induced toxicity. Nevertheless, it is alleged that the administration of antioxidants along with anticancer agents may help relieve the toxic side effects elicited by these agents. Consequently, the present study was designed to investigate the protective effect of quercetin pretreatment and cotreatment on melphalan-induced renal and hepatic toxicity in rat models.

## 2. Materials and Methods

### 2.1. Chemicals and Reagents

Melphalan tablets were a product of Excella GmbH, Nürnberger, Germany. Quercetin, glutathione, 1-chloro-2,4-dinitrobenzene (CDNB), 5,5-dithiobis-2-nitrobenzoic acid (DTNB), epinephrine, and hydrogen peroxide (H_2_O_2_) were all purchased from Sigma Chemical Company (London, UK). Kits for alanine transferase (ALT), aspartate aminotransferase (AST), alkaline phosphatase (ALP), gamma glutamyl transpeptidase (GGT), urea, creatinine, and total bilirubin were products of Randox laboratories Ltd. (Antrim, UK). All other reagents used were of analytical grade and of the highest purity.

### 2.2. Animal Selection and Care

Twenty-five male Wistar rats weighing between 160 and 170 g were obtained from The Animal Holding Unit, Department of Chemical Sciences, Ajayi Crowther University, Oyo, Nigeria. The rats were acclimatized under laboratory conditions prior to experiment. The animals were housed in wire-meshed cages and provided with food and water* ad libitum*. The animals were maintained under standard conditions of temperature and humidity with 12-hour light/dark cycles. They were fed with commercial rat diet (Ladokun Feeds, Nigeria Ltd.). Handling of the experimental animals was in conformity with the National Institutes of Health's Guide for the Care and Use of Laboratory Animals (NIH publication number 85-23 revised 1985; US Department of Health, Education and Welfare, Bethesda, MA).

### 2.3. Animal Grouping and Drug Treatments

The animals were randomly assigned into five experimental groups (I–V) of five animals each. The animals of each group were treated as presented in Table 1. The doses for MPLN and QCT were decided based on the available literature [32, 33] and were delivered in one mL solution of distilled water once daily by oral intubation.

### 2.4. Animal Sacrifice and Collection of Blood and Liver Samples

Blood samples were collected from each animal through retro orbitals plexus into heparinized tubes (Li heparin). Animals were sacrificed and the liver was carefully excised from each animal for preparation of cytosolic fraction.

### 2.5. Preparation of Plasma and Cytosolic Fractions

Plasma was obtained by centrifugation of whole blood sample at 4000 rpm, for 5 minutes using a Cencom bench centrifuge. The plasma obtained was stored at −4°C for subsequent plasma assays. The liver excised from each rat was blotted of blood stains, rinsed in ice-cold 1.15% KCl, and homogenized in 4 volumes of ice-cold 0.01 M potassium phosphate buffer (pH 7.4). The homogenates were centrifuged at 12,500 g for 15 min at −4°C (Eppendorf, UK) and the supernatants, termed the postmitochondrial fractions (PMF), were aliquoted and used for subsequent biochemical assays.

### 2.6. Determination of Plasma and Liver Protein Content

The protein concentration in the plasma and liver homogenate was determined by the Biuret method of Gornall et al. [34] using bovine serum albumin as standard.

### 2.7. Assay of Plasma Biomarkers of Renal Toxicity

Plasma urea and creatinine were determined with Randox diagnostic kits. Method for creatinine assays was based on colorimetric alkaline picrate methods [35] with creatinine-picrate complex measured at 492 nm. Plasma urea determination was based on the Fenton reaction [36] with the diazine chromogen formed absorbing strongly at 540 nm.

### 2.8. Assay of Plasma Biomarkers of Hepatotoxicity

Plasma total bilirubin (TBILI) determination was done using Randox diagnostic kits based on the dimethyl sulphoxide method by Tietz [36]. The dimethyl sulphoxide forms a coloured compound with maximum absorption at 550 nm. Plasma alkaline phosphatase (ALP), alanine aminotransferase (ALT), aspartate aminotransferase (AST), and gamma glutamyl transferase (*γ*-GT) activities were determined using Randox diagnostic kits. ALP activity was determined in accordance with the principles of Tietz et al. [37]. The p-nitrophenol formed by the hydrolysis of p-nitrophenyl phosphate confers yellowish colour on the reaction mixture and its intensity can be monitored at 405 nm to give a measure of enzyme activity. Determination of plasma ALT and AST activities was based on the principle described by Reltman and Frankel [38]. ALT activity was measured by monitoring the concentration of pyruvate hydrazone formed with 2,4-dinitrophenylhydrazine at 546 nm. AST activity was measured by monitoring the concentration of oxaloacetate hydrazone formed with 2,4-dinitrophenylhydrazine at 546 nm. *γ*-GT activity was determined following the principle described by Szasz [39]. The substrate L-*γ*-glutamyl-3-carboxy-4-nitroanilide, in the presence of glycylglycine, is converted to 5-amino-2-nitrobenzoate by *γ*-GT measured at 405 nm. The increase in absorbance is proportional to *γ*-GT activity.

### 2.9. Assay for Nonenzymatic Antioxidants in the Liver

Hepatic reduced glutathione level was determined according to the method of Jollow et al. [40]. The chromophoric product resulting from the reaction of Ellman's reagent with the reduced glutathione, 2-nitro-5-thiobenzoic acid, possesses a molar absorption at 412 nm which was read in a spectrophotometer. Reduced GSH is proportional to the absorbance at 412 nm. The ascorbic acid concentration was determined according to the method of Jagota and Dani [41]. AA in biological samples reacts with Folin's reagent, an oxidizing agent to give a blue color which has its maximum absorption at 760 nm.

### 2.10. Assay of Hepatic Antioxidant Enzymes

Hepatic glutathione S-transferase (GST) activity was determined by the method described by Habig et al. [42] using 1-chloro-2,4-dinitrobenzene (CDNB) as substrate. The procedure of Misra and Fridovich [43] was used for the determination of hepatic superoxide dismutase (SOD) activity by measuring the inhibition of autooxidation of epinephrine at pH 10.2 and 30°C. Hepatic catalase activity was determined by the method described by Sinha [44] based on the reduction of dichromate in acetic acid to chromic acetate when heated in the presence of hydrogen peroxide (H_2_O_2_). The chromic acetate produced is measured spectrophotometrically at 570 nm.

### 2.11. Assay of Hepatic Level of Lipid Peroxidation

The extent of lipid peroxidation (LPO) in the liver was estimated by the method of Varshney and Kale [45]. The method involved the reaction between malondialdehyde (MDA; product of lipid peroxidation) and thiobarbituric acid to yield a stable pink chromophore with maximum absorption at 532 nm.

### 2.12. Statistical Analysis

The results were expressed as means of 5 replicates ± SD. Data obtained were subjected to one-way analysis of variance (ANOVA) followed by Duncan multiple range test for comparison between control and treated rats in all groups using* SigmaPlot* statistical application package. *P* values less than 0.05 were considered statistically significant.

## 3. Results

### 3.1. Plasma Biomarkers of Renal Toxicity

Table 2 shows the protective effects of QCT on MPLN-induced changes in plasma creatinine and urea in rats. Administration of MPLN caused a significant increase in the plasma level of creatinine and urea by 188% and 102.3%, respectively, when compared with the control. Pre- and cotreatment with QCT significantly protected against the increase in plasma level of creatinine and urea in rats.

### 3.2. Plasma Biomarkers of Hepatotoxicity

Plasma bilirubin level reduced by 122.2% following MPLN administration (Table 2). Similar trends were also observed for the activities of the liver enzymes, ALT, AST, ALP, and *γ*-GT, in the plasma of experimental animals. The activities of ALT, AST, ALP, and *γ*-GT in the plasma of MPLN-treated rats increased significantly by 144.3%, 71.3%, 57.9%, and 307.2%, respectively, compared to the values of the control animals (Table 3). However, quercetin pre- and cotreatment significantly ameliorated the MPLN-induced increase in plasma bilirubin as well as plasma ALT, AST, ALP, and *γ*-GT in rats.

### 3.3. Activity of Antioxidant Enzymes (SOD, CAT, and GST)

Table 4 represents the protective effect of quercetin on MPLN-induced reduction in the activities of SOD and CAT in the liver of rats. Hepatic SOD and CAT activities were significantly reduced in the MPLN-treated group by 34.4% and 52.2%, respectively, when compared with values of the control group. Hepatic GST activity was also significantly reduced by 46.5% when compared to control (Figure 4). However, pretreatment and cotreatment with quercetin significantly ameliorated the activities of hepatic SOD, CAT, and GST.

### 3.4. Nonenzymic Antioxidants (AA and GSH)

**Figure 2:**
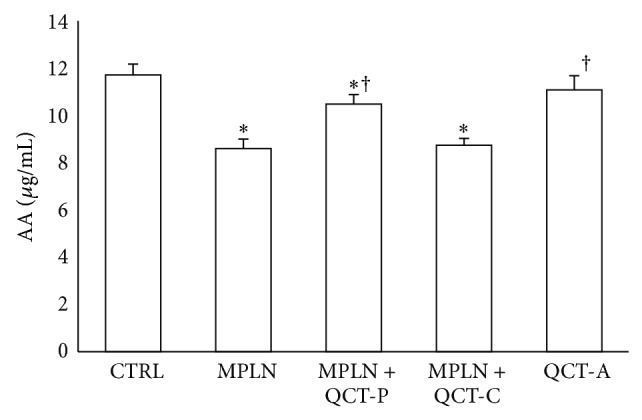
Protective effects of quercetin on melphalan-induced changes in hepatic AA level in rats. Data represent the means ± SD for five rats in each group;  ^*^significantly different from the CTRL;  ^†^significantly different from MPLN.

**Figure 3:**
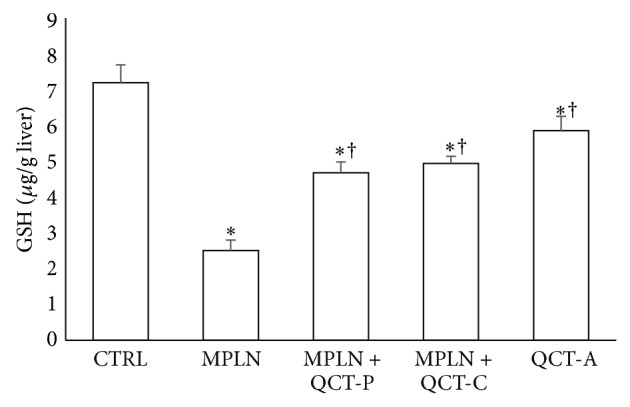
Protective effect of quercetin on melphalan-induced changes in the levels of GSH in rats. Data represent the means ± SD for five rats in each group;  ^*^significantly different from the CTRL;  ^†^significantly different from MPLN.

**Figure 4:**
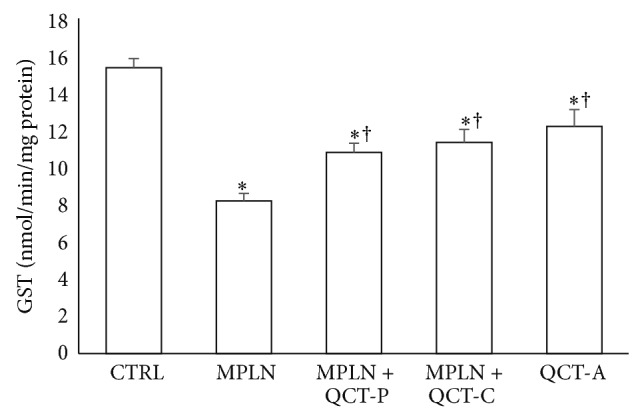
Protective effect of quercetin on melphalan-induced changes in hepatic GST activity in rats. Data represent the means ± SD for five rats in each group;  ^*^significantly different from the CTRL;  ^†^significantly different from MPLN.

Hepatic AA level also reduced significantly by 36.2% following oral administration of MPLN to rats (Figure 2). A similar decrease in hepatic GSH (by 188%) was also observed (Figure 3) in the MPLN-treated animals relative to control. The level of these nonenzymatic antioxidants was enhanced following pre- and cotreatment with quercetin.

### 3.5. Lipid Peroxidation

**Figure 5:**
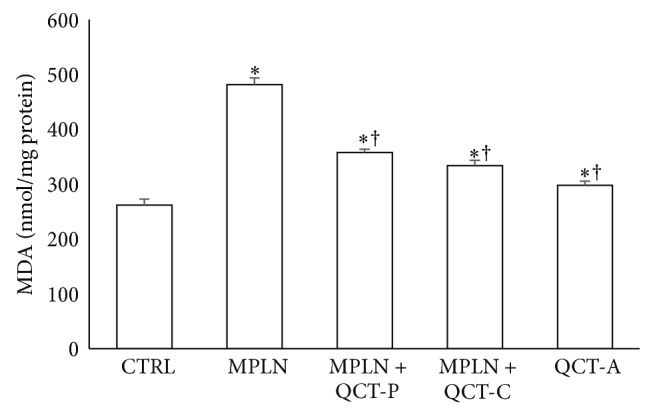
Protective effect of quercetin on melphalan-induced changes in the level of lipid peroxidation (MDA) in rats. Data represent the means ± SD for five rats in each group;  ^*^significantly different from the CTRL;  ^†^significantly different from MPLN.

The MPLN-induced reduction in hepatic antioxidant status was accompanied by a significant increase in the level of lipid peroxidation (as indicated by the MDA content), as shown in Figure 5. The level of lipid peroxidation was significantly increased in MPLN-treated group by 46.5% when compared with the control. Pretreatment and cotreatment with quercetin significantly attenuated this increase in hepatic MDA when compared with melphalan group.

## 4. Discussion

The present study evaluates renal and hepatotoxic effect of melphalan (MPLN) in Wistar rats and possible protection by the flavonoid antioxidant, quercetin (QCT). The plasma biomarkers of renal function, creatinine and urea, were considered in this study. Creatinine and urea are metabolic products which are removed from circulation by the kidney to prevent their accumulation. Increase in plasma level of these substances is regarded as an indication of loss of renal function [46, 47]. Data from this study suggest that alkylating agents caused a loss of renal function and this is consistent with previous reports [48, 49]. We observed that QCT ameliorated the levels of plasma creatinine and urea which is an indication of renal protection. This also confirms the protective role of QCT against drug-induced renal toxicity as previously reported [50, 51]. The liver is an organ involved in the biotransformation of drugs and other hepatotoxicants. The plasma level of bilirubin and activities of the liver enzymes, ALT, AST, ALP, and *γ*-GT, are considered reliable indices of hepatotoxicity [52, 53]. In this study, MPLN caused a significant increase in the plasma bilirubin level and activities of ALT, AST, ALP, and *γ*-GT. ALT and AST are primarily located in the cytoplasm and mitochondria of hepatocytes [54]. Increase in plasma ALT and AST may have resulted from leakage from damaged hepatocytes (hepatocellular injury) [55]. Bilirubin is found in the liver, bile, intestines, and the reticuloendothelial cells of the spleen while ALP and *γ*-GT are associated with the cell membrane [56]. Plasma bilirubin and activities of ALP and *γ*-GT are found to increase in conditions associated with hepatobiliary injury (decrease in hepatic clearance of bilirubin) and overproduction or leakage of ALP and *γ*-GT [56]. In this study, pre- and cotreatment with quercetin protected against increase in plasma levels of bilirubin, ALT, AST, ALP, and *γ*-GT, which is an indication of hepatoprotection by QCT. Our observation also corroborates previous findings showing the hepatoprotective activity of QCT [30, 57].

Several studies have established a connection between hepatotoxicity and oxidative stress [58–60], thus prompting the consideration of the effect of MPLN on major enzymic as well as nonenzymic antioxidant systems of rats. Activities of enzymic antioxidants, SOD, CAT, and GST, are vital to the maintenance of the cellular redox balance [61]. In this study, MPLN significantly decreases the activity of SOD, CAT, and GST in the liver of rats. SOD catalyzes the reaction involving a rapid dismutation of superoxide radical to hydrogen peroxide and dioxygen while CAT converts the hydrogen peroxide formed in this process and other cellular processes into water and molecular oxygen [62]. Reduction in the activities of SOD and CAT by MPLN may expose the liver to oxidative stress [37]. Reduction in hepatic SOD activity is an indication of oxidative stress [51]. Similarly, decrease in the activity of CAT in the liver of MPLN rats may have resulted from accumulation of superoxide anion radical due to reduction in hepatic SOD activity [52]. GST is an enzyme found in most tissues and it is involved in the detoxification of ingested xenobiotics in the liver [63, 64] and also forms a vital component of the antioxidant defense mechanism [64, 65].

The non-enzymic antioxidant molecules, AA and GSH, play a crucial role in cellular redox balance. Both AA and GSH are involved in scavenging ROS and are the first line of defense against oxidation [15]. AA functions in the aqueous environments of the body and is involved in the regeneration of tocopherol from tocopherol radicals in membranes and lipoproteins [66, 67]. One of the major roles of glutathione against oxidative stress includes acting as a cofactor for several enzymic antioxidants like glutathione peroxidase (GPx), glutathione-S-transferase, and others, also scavenging hydroxyl radical and singlet oxygen and regeneration of other antioxidants such as vitamins C and E back to their active forms [68]. Disturbance in the cellular redox status of AA and GSH has been reported to enhance oxidative stress and tissue injury [46]. Pre- and cotreatment with QCT significantly improved the levels of AA and GSH in rats which supports previous reports by Mishra et al. [30] and Dong et al. [31].

Increase in tissue MDA content (from oxidation of unsaturated fatty acids) is a commonly used marker of oxidative stress [15]. Lipid peroxidation is initiated by the attack of a free radical on fatty acid [18] and leads to cell and tissue damage. The observed significant increase in the concentration of MDA in the liver of MPLN-treated animals may be related to decreased antioxidant protection from free radicals [31].

## 5. Conclusion

In conclusion, we report that QCT has the capacity to protect against MPLN-induced hepatotoxicity and oxidative stress probably through scavenging the free radicals.

## Figures and Tables

**Table 1:** Experimental design.

Treatment ↓groups	Treatments	
Duration →	Days 1–7	Days 8–14
I (CTRL)	—	Control; distilled water
II (MPLN)	—	0.2 mg/Kg b.w. MPLN
III (MPLN + QCT-P)	20 mg/Kg b.w. QCT	0.2 mg/Kg b.w. MPLN
IV (MPLN + QCT-C)	—	0.2 mg/Kg b.w. MPLN + 20 mg/Kg b.w. QCT
V (QCT-A)	—	20 mg/Kg b.w. QCT

CTRL: control, MPLN: melphalan, QCT: quercetin, QCT-P: quercetin-pretreated, QCT-C: quercetin-cotreated, QCT-A: quercetin-alone, b.w.: body weight.

**Table 2:** Protective effects of quercetin on melphalan-induced changes in the levels of plasma creatinine, urea, and bilirubin levels in rats.

Treatment	Creatinine (mg/dL)	Urea (mg/dL)	Bilirubin (mg/dL)
CTRL	0.98 ± 0.13	51.36 ± 0.23	0.36 ± 0.01
MPLN	2.14 ± 0.11 (188%)^*^	103.9 ± 0.19 (102.3%)^*^	0.80 ± 0.02 (122.2%)^*^
MPLN + QCT-P	1.76 ± 0.09^∗†^	76.9 ± 0.64^∗†^	0.55 ± 0.02^∗†^
MPLN + QCT-C	1.52 ± 0.08^∗†^	95.8 ± 0.84^∗†^	0.51 ± 0.01^∗†^
QCT-A	1.38 ± 0.08^∗†^	74.2 ± 1.30^∗†^	0.46 ± 0.01^∗†^

Data represent the means ± SD for five rats in each group; ^*^significantly different from the CTRL; ^†^significantly different from MPLN. Values in parenthesis represent percentage (%) increase.

**Table 3:** Protective effects of quercetin on melphalan-induced changes in the plasma activities of ALT, AST, ALP, and *γ*-GT in rats.

Treatment	ALT (U/L)	AST (U/L)	ALP (U/L)	*γ*-GT (U/L)
CTRL	21 ± 0.24	174 ± 2.30	257 ± 2.41	1.4 ± 0.16
MPLN	45 ± 1.22 (144.3%)^*^	298 ± 3.32 (71.3%)^*^	406 ± 2.61 (57.9%)^*^	5.7 ± 0.28 (307.2%)^*^
MPLN + QCT-P	34 ± 1.40^∗†^	204 ± 2.30^∗†^	319 ± 2.28^∗†^	4.5 ± 0.23^∗†^
MPLN + QCT-C	28 ± 0.56^∗†^	195 ± 3.87^∗†^	331 ± 2.28^∗†^	4.2 ± 0.25^∗†^
QCT-A	27 ± 0.74^∗†^	184 ± 1.59^∗†^	279 ± 2.28^∗†^	3.2 ± 0.19^∗†^

Data represent the means ± SD for five rats in each group; ^*^significantly different from the CTRL; ^†^significantly different from MPLN. Values in parenthesis represent percentage (%) increase.

**Table 4:** Protective effect of quercetin on melphalan-induced changes in hepatic SOD and CAT activities in rats.

Treatment	SOD (Units)	CAT (*μ*mole H_2_O_2_ consumed/min/mg protein)
CTRL	8.38 ± 0.18	0.23 ± 0.02
MPLN	5.5 ± 0.22 (34.4%)^*^	0.11 ± 0.01 (52.2%)^*^
MPLN + QCT-P	7.04 ± 0.17^∗†^	0.19 ± 0.01^∗†^
MPLN + QCT-C	7.48 ± 0.18^∗†^	0.18 ± 0.01^∗†^
QCT-A	7.84 ± 0.11^∗†^	0.22 ± 0.02^*^

Data represent the means ± SD for five rats in each group; ^*^significantly different from the CTRL; ^†^significantly different from MPLN. Values in parenthesis represent percentage (%) decrease.
